# Integrated analysis of lncRNA-miRNA-mRNA ceRNA network in squamous cell carcinoma of tongue

**DOI:** 10.1186/s12885-019-5983-8

**Published:** 2019-08-07

**Authors:** Rui-Sheng Zhou, En-Xin Zhang, Qin-Feng Sun, Zeng-Jie Ye, Jian-Wei Liu, Dai-Han Zhou, Ying Tang

**Affiliations:** 1 The First Affiliated Hospital of Guangzhou University of Chinese Medicine, Guangzhou University of Chinese Medicine, Guangzhou, China; 2 Stomatological Hospital of Shandong University, Shandong, China; 3 Guangzhou University of Chinese Medicine, Guangzhou, China; 4 Jinan stomatological hospital, Shandong, China; 5 Lingnan Medical Research Center of Guangzhou University of Chinese Medicine, Guangzhou, China

**Keywords:** Squamous cell carcinoma of the tongue, Long non-coding RNAs, Competing endogenous RNAs network, The Cancer genome atlas, Overall survival

## Abstract

**Background:**

Numerous studies have highlighted that long non-coding RNAs (lncRNAs) can bind to microRNA (miRNA) sites as competing endogenous RNAs (ceRNAs), thereby affecting and regulating the expression of mRNAs and target genes. These lncRNA-associated ceRNAs have been theorized to play a significant role in cancer initiation and progression. However, the roles and functions of the lncRNA-miRNA-mRNA ceRNA network in squamous cell carcinoma of the tongue (SCCT) are still unclear.

**Methods:**

The miRNA, mRNA and lncRNA expression profiles from 138 patients with SCCT were downloaded from The Cancer Genome Atlas database. We identified the differential expression of miRNAs, mRNAs, and lncRNAs using the limma package of R software. We used the clusterProfiler package for GO and KEGG pathway annotations. The survival package was used to estimate survival analysis according to the Kaplan-Meier curve. Finally, the GDCRNATools package was used to construct the lncRNA-miRNA-mRNA ceRNA network.

**Results:**

In total, 1943 SCCT-specific mRNAs, 107 lncRNAs and 100 miRNAs were explored. Ten mRNAs (CSRP2, CKS2, ADGRG6, MB21D1, GMNN, RIPOR3, RAD51, PCLAF, ORC1, NAGS), 9 lncRNAs (LINC02560, HOXC13 − AS, FOXD2 − AS1, AC105277.1, AC099850.3, STARD4 − AS1, SLC16A1 − AS1, MIR503HG, MIR100HG) and 8 miRNAs (miR − 654, miR − 503, miR − 450a, miR − 379, miR − 369, miR − 190a, miR − 101, and let−7c) were found to be significantly associated with overall survival (log-rank *p* < 0.05). Based on the analysis of the lncRNA-miRNA-mRNA ceRNA network, one differentially expressed (DE) lncRNA, five DEmiRNAs, and three DEmRNAs were demonstrated to be related to the pathogenesis of SCCT.

**Conclusions:**

In this study, we described the gene regulation by the lncRNA-miRNA-mRNA ceRNA network in the progression of SCCT. We propose a new lncRNA-associated ceRNA that could help in the diagnosis and treatment of SCCT.

**Electronic supplementary material:**

The online version of this article (10.1186/s12885-019-5983-8) contains supplementary material, which is available to authorized users.

## Background

Head and neck squamous cell carcinoma (HNSCC), which is a disease that causes serious harm to humans, is highly correlated with alcohol consumption, tobacco smoking, and betel nut chewing, and human papillomavirus infection. Squamous cell carcinoma of the tongue (SCCT) is a particular subtype and the main cause of patient mortality and morbidity from HNSCC [1, 2]. In general, the clinical features and treatment strategies for SCCT are similar to those of other HNSCCs, with surgical resection being the primary treatment choice. However, due to late diagnosis of locally advanced malignancies, in many cases of SCCT, surgery is either no longer an option, or should be avoided to maintain the patient’s quality of life [3, 4]. Despite the advances in treatment options, the prognosis of patients with advanced SCCT remains poor [5]. In China, although pingyangmycin and/or cisplatin-based chemotherapies have shown good results, chemotherapy resistance always develops later and causes the therapy to fail [6]. In the past three decades, the 5-year survival rate of patients with SCCT was less than 50% [7]. Therefore, the main goal of our research has been to obtain more knowledge about SCCT cells and to identify novel therapeutic targets for treating the disease.

Long non-coding RNAs (lncRNAs), which do not have protein-coding functions, have recently attracted increasing research attention [8, 9]. These RNAs play a significant role in different cellular processes, particularly in numerous kinds of tumors [10–12]. For example, lncRNAs can act as biomarkers for the prognosis and diagnosis of lung adenocarcinoma [13]. MicroRNAs (miRNAs) are small, endogenous, non-coding RNAs composed of 19–25 nucleotides [14, 15]. They exert the important function of regulating gene expression, and their regulatory networks are involved in many biological processes [16–18]. In 2011, Salmena et al. proposed the competitive endogenous RNA (ceRNA) hypothesis [19], which was subsequently supported by several lines of evidence [20–24]. This hypothesis describes the competitive activity of some RNAs (as ceRNAs) for common binding sites of target miRNAs, thereby altering the function of the target miRNA [25]. The core concept is that ceRNAs interact with target miRNAs through miRNA response elements to control the transcriptome on a large scale. In the past several years, lncRNAs and SCCT were confirmed to be closely related. For instance, expression of the lncRNA SNHG6 is significantly increased in tongue cancer, and interference with SNHG6 expression can inhibit the proliferation and epithelial–mesenchymal transition (EMT) of tongue cancer cells [26]. Zhang et al. found that the oncogenic lncRNA KCNQ1OT1 plays a vital role in SCCT growth and chemoresistance, and can be used as a new target for SCCT treatment [27]. However, previous studies had focused on the mechanism of a single lncRNA-miRNA-mRNA axis, and there is currently no reported ceRNA network in SCCT. Consequently, it is extremely important to investigate the role of ceRNA networks in the poor prognosis of SCCT. By further learning how lncRNAs function in the pathogenesis of SCCT, we may find solutions to the most pressing challenges faced in treating this disease.

In this study, the mRNA, miRNA, and lncRNA expression profiles of SCCT and normal tissues were downloaded from The Cancer Genome Atlas (TCGA). In addition, through comprehensive analysis, the ceRNA network for SCCT was builted, which will serve to find new targets and pathways for the development of treatments to prolong patient survival times. Finally, we conducted a prognostic analysis with several important lncRNAs and found a biomarker that could predict survival in patients with SCCT.

## Methods

### Patients and samples

The SCCT cases data of clinical and RNA expression were collected from TCGA database. The exclusion criteria were including: (i) histological diagnosis was not SCCT; (ii) no complete data (including gender, age, survival status, stage, and survival time) for analysis [28]. 118 SCCT patients were enrolled in the study. The number of patients aged < 68 years was 49, 69 patients were ≥ 68 years old. 43 patients were female and 75 patients were male. The number of stage I, II, III, IVa and IVb patients were 13, 20, 27, 56 and 2, respectively. The number of patients, who were white, Asian, black or African American and not available, were 106, 5, 5 and 2, respectively. The number of patients, who were hispanic or latino, were 104. 9 patients were not hispanic or latino, and 5 patients were not reported. 46 patients were dead, and 72 patients were alive. SCCT characteristics and clinical data of the patients are showed in Table 1 and Additional file 3: Table S1.

**Table 1:** 118 tongue squamous cell carcinoma patients characteristics and clinical data

Characteristics	N (%)
Age (year) (mean ± SD)	68.66 ± 14.44
< 68	49 (41.53)
≧68	69 (58.47)
Sex	
Male	75 (63.56)
Female	43 (36.44)
Race	
White	106 (89.83)
Asian	5 (4.24)
Black or african american	5 (4.24)
Not available	2 (1.69)
Ethnicity	
not hispanic or latino	9 (7.63)
hispanic or latino	104 (88.13)
not reported	5 (4.24)
Tumor stage	
I	13 (11.02)
II	20 (16.95)
III	27 (22.88)
IVa	56 (47.46)
IVb	2 (1.69)
Survival status	
Dead	46 (38.98)
Alive	72 (61.02)

### RNA sequence analysis

RNA expression data of SCCT patients were available from TCGA database. The raw reads of lncRNA and mRNA data were post-treated and normalized in R software (Additional file 1: Figure S1). The miRNA expression data from TCGA database were normalized in R software (Additional file 2: Figure S2). The tumor tissue and adjacent non-tumor tissue of SCCT patients were facilitated differential expressions of mRNA, lncRNA, and miRNA. Furthermore, intersection of lncRNA, miRNA and mRNA was selected [13].

### Differentially expressed analysis

Compared to the normal group with SCCT, “limma” package in R software was used to identify the differentially expressed mRNAs (DEmRNAs) with thresholds of |fold Change (FC)| > 2.0 and *P* value < 0.01 and differentially expressed miRNAs with |FC| > 2.5 and *P* value < 0.01.

### Functional enrichment analysis

“ClusterProfiler” package in R software was used for functional enrichment analysis, and GO biological processes and KEGG pathways at the significant level (q-value < 0.01) were employed.

### Survival analysis

To determine the prognostic characteristics of DERNAs, combining the clinical data the survival curves of these samples with differentially expressed mRNA, lncRNA and miRNA were plotted by using the “survival” package in R based on Kaplan-Meier curve analysis. *P* values < 0.05 were regarded as significant.

### Construction of lncRNA-miRNA-mRNA ceRNA network

The lncRNA-miRNA-mRNA ceRNA network was based on the theory that lncRNAs can directly interact by invoking miRNA sponges to regulate mRNA activity [29]. “GDCRNATools” (http://bioconductor.org/packages/devel/bioc/html/GDCRNATools.html) package in R software were used to establish ceRNA network [30]. The ceRNA network was plotted with Cytoscape v3.6.0 [31]. The plug-in BinGO of Cytoscape is an APP for BF network of the hub genes [32].

## Results

### Identification of differentially expressed lncRNA, miRNA and mRNA

**Fig. 1:**
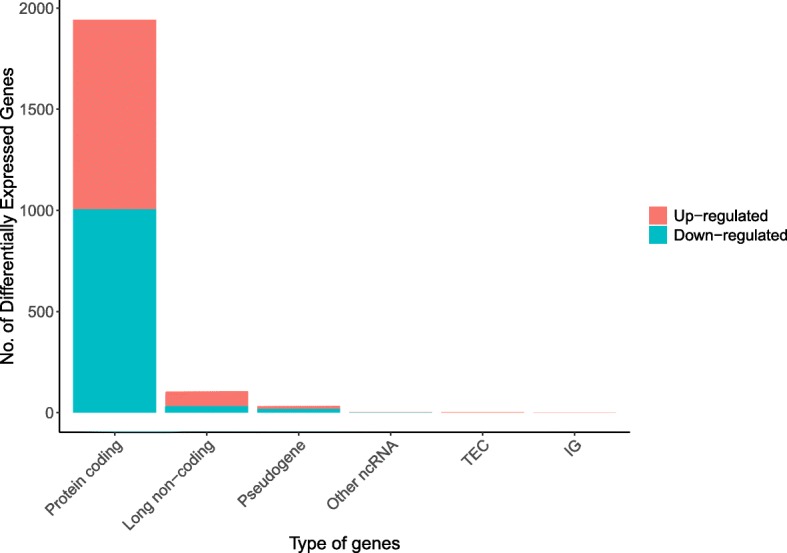
Column diagram of DEGs DEGs were selected with thresholds of fold change > 2 and *p* < 0.01.

**Fig. 2:**
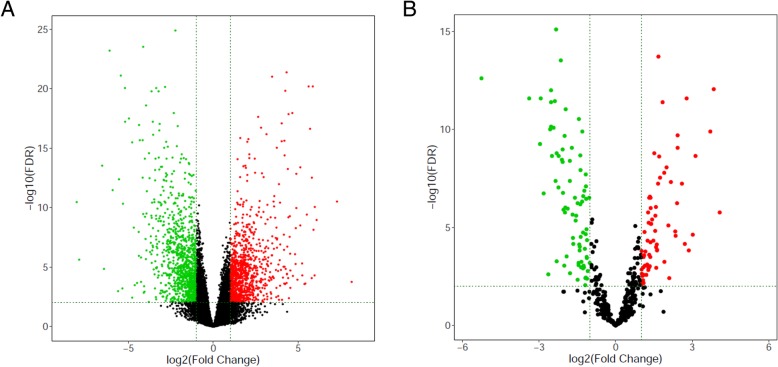
Volcano Plot of DEGs and DEmRNAs. **a** Volcano Plot of DEGs. **b** Volcano Plot of differentially expressed miRNA. Upregulated genes are marked in light red; downregulated genes are marked in light green. (DEGs were selected with thresholds of fold change > 2 and *p* < 0.01, DEmRNAs were selected with thresholds of fold change > 2.5 and *p* < 0.01)

We explored 1943 SCCT-specific mRNAs (1007 downregulated and 936 upregulated; Table 2 and Fig. 1) and 107 lncRNAs (34 downregulated and 73 upregulated; Fig. 1, Table 2, and Table 3). The differentially expressed genes (DEGs) are shown in Fig. 2a. Additionally, 100 miRNAs (44 upregulated and 56 downregulated; Fig. 2b, and Table 4) were found.

**Table 2:** Top 20 up-regulated mRNAs and lncRNAs

Top 20 up-regulated mRNAs			
mRNA	LogFC	*P*-Value	FDR
TGFBI	4.315537595	1.17E-25	4.22E-22
PLAU	3.462663797	4.15E-25	9.92E-22
LAMC2	4.237080843	1.28E-23	1.54E-20
HOXC6	4.65525797	1.36E-21	1.15E-18
HOXA1	4.433954775	1.64E-21	1.31E-18
SERPINH1	2.639882646	3.24E-21	2.45E-18
COL4A2	2.81823357	3.44E-20	1.83E-17
HOXC11	5.695015733	4.79E-20	2.46E-17
COL4A1	3.133084242	1.36E-19	6.51E-17
COLGALT1	1.58936638	3.17E-19	1.42E-16
FSCN1	2.073990216	4.00E-19	1.74E-16
COL1A1	4.185143619	5.70E-19	2.27E-16
COL5A1	4.027658252	7.17E-19	2.78E-16
PTK7	2.001886655	8.17E-19	3.01E-16
COL12A1	3.740382035	2.85E-18	9.30E-16
MYO1B	2.125906022	1.10E-17	3.36E-15
HOXC4	4.197008216	1.40E-17	4.18E-15
CD276	2.126335416	2.60E-17	7.18E-15
BMP1	2.44274785	2.87E-17	7.64E-15
PPP1R18	1.435370823	3.51E-17	8.55E-15
Top 20 up-regulated lncRNAs			
lncRNA	LogFC	P-Value	FDR
AL358334.2	5.609527114	3.12E-24	6.30E-21
LINC02081	5.861313467	3.51E-24	6.30E-21
AC114956.2	4.026119266	1.38E-20	8.64E-18
LINC00941	4.887474201	8.07E-16	1.47E-13
AC002384.1	5.807235765	1.80E-15	2.90E-13
ZFPM2-AS1	5.506777453	5.05E-12	3.84E-10
LINC01615	4.417820628	9.78E-12	6.69E-10
GSEC	2.402552017	3.38E-11	2.02E-09
LINC01322	5.896783476	1.57E-10	7.73E-09
AL024507.2	1.883167255	2.54E-10	1.17E-08
AL365356.5	3.477166051	6.36E-10	2.58E-08
FOXD2-AS1	2.111692628	7.75E-10	3.05E-08
MYOSLID	3.218244344	1.68E-09	5.85E-08
TM4SF19-AS1	2.540840853	1.80E-08	4.44E-07
MIR503HG	2.967470865	2.81E-08	6.42E-07
AC009948.1	1.233483122	2.81E-08	6.42E-07
AC099850.3	2.086563885	6.07E-08	1.25E-06
AC012073.1	1.49221094	6.69E-08	1.36E-06
U62317.2	3.239685972	7.99E-08	1.58E-06
LINC01116	2.92964907	1.23E-07	2.31E-06

**Table 3:** Top 20 down-regulated mRNAs and lncRNAs

Top 20 down-regulated mRNAs			
mRNA	LogFC	*P*-Value	FDR
CAB39L	−2.230860951	8.79E-30	1.26E-25
SH3BGRL2	−4.129195634	4.15E-28	2.98E-24
FAM3D	−6.109712255	1.31E-27	6.29E-24
FUT6	−5.441041935	2.63E-25	7.54E-22
GPD1L	−2.833273831	4.33E-24	6.91E-21
CYP4B1	−5.201620597	5.94E-24	8.53E-21
SELENBP1	−3.356944126	6.87E-24	8.97E-21
TLE2	−3.218328165	1.65E-23	1.75E-20
CGNL1	−3.633348282	1.70E-23	1.75E-20
HLF	−3.950815219	2.57E-22	2.47E-19
PAIP2B	−2.325857173	1.31E-21	1.15E-18
FMO2	−4.962551951	4.23E-21	3.04E-18
TF	−5.192763131	9.20E-21	6.23E-18
RORC	−3.561163029	9.54E-21	6.23E-18
DEPTOR	−3.156822796	1.61E-20	9.61E-18
PLIN4	−4.374193766	2.01E-20	1.16E-17
AGFG2	−2.096127174	2.62E-20	1.45E-17
RRAGD	−3.179962858	6.47E-20	3.21E-17
FAM107A	−3.539763631	1.69E-19	7.81E-17
ALDH1A1	−4.321886949	5.20E-19	2.20E-16
Top 20 down-regulated lncRNAs			
lncRNA	LogFC	*P*-Value	FDR
ZNF710-AS1	−2.097998239	1.42E-13	1.64E-11
AC104825.2	−1.895549481	2.81E-12	2.27E-10
C5orf66	−1.666569905	9.75E-11	5.11E-09
AL035661.1	−2.362499591	1.75E-10	8.45E-09
WFDC21P	−2.838242509	4.55E-10	1.94E-08
AL691432.2	−1.328157969	5.37E-09	1.57E-07
CBR3-AS1	−1.384547792	1.41E-08	3.61E-07
DANCR	−1.479846155	1.49E-08	3.78E-07
LINC00957	− 1.501952779	1.84E-08	4.50E-07
AC144831.1	−1.8197168	1.92E-08	4.67E-07
EPB41L4A-AS1	−1.119545231	1.97E-08	4.81E-07
AC009506.1	−1.117948729	9.68E-08	1.86E-06
AC068888.1	−1.291110108	3.26E-07	5.29E-06
ZNF667-AS1	−1.77757381	4.94E-07	7.43E-06
AL357033.4	−1.625781202	6.24E-07	9.13E-06
AC023283.1	−1.450946619	8.16E-07	1.14E-05
AL109976.1	−1.516544927	3.93E-06	4.27E-05
SPINT1-AS1	−1.334348352	4.73E-06	4.98E-05
CEBPA-AS1	−1.077762786	1.62E-05	0.000139196
LINC01133	−2.012971887	2.62E-05	0.000209882

**Table 4:** Differentially expressed miRNAs (Top 40)

Top 20 up-regulated miRNAs			
miRNA	LogFC	*P*-Value	FDR
hsa-miR-21-5p	1.679798023	7.95E-17	1.87E-14
hsa-miR-615-3p	3.843177883	9.14E-15	8.61E-13
hsa-miR-455-3p	2.783479203	4.93E-14	2.61E-12
hsa-miR-1301-3p	1.837395098	1.08E-13	4.23E-12
hsa-miR-196b-5p	3.706120237	5.20E-12	1.29E-10
hsa-miR-424-3p	2.425887565	8.59E-12	2.02E-10
hsa-miR-877-5p	2.426291524	4.51E-11	8.84E-10
hsa-miR-21-3p	1.51590429	9.61E-11	1.68E-09
hsa-miR-503-5p	3.129677768	1.40E-10	2.27E-09
hsa-miR-2355-5p	1.702296959	1.70E-10	2.51E-09
hsa-miR-2355-3p	1.998917306	6.52E-10	8.53E-09
hsa-miR-450a-5p	1.904455832	1.38E-09	1.71E-08
hsa-miR-424-5p	1.738999807	2.45E-09	2.88E-08
hsa-miR-224-5p	2.165626373	4.47E-09	4.90E-08
hsa-miR-503-3p	2.595299327	5.43E-09	5.82E-08
hsa-miR-671-5p	1.663707163	5.65E-09	5.92E-08
hsa-miR-1307-3p	1.345431238	3.09E-08	2.80E-07
hsa-miR-130b-5p	1.368955234	3.42E-08	3.04E-07
hsa-miR-365a-5p	2.405326455	7.33E-08	5.85E-07
hsa-miR-193b-3p	1.581297442	1.22E-07	9.30E-07
Top 20 down-regulated miRNAs			
miRNA	LogFC	*P*-Value	FDR
hsa-miR-101-3p	−2.332600896	1.63E-18	7.69E-16
hsa-miR-30a-5p	−2.147092552	1.97E-16	3.10E-14
hsa-miR-375	−5.251608936	2.17E-15	2.55E-13
hsa-miR-30a-3p	−2.529446797	1.27E-14	9.94E-13
hsa-miR-99a-5p	−2.931395278	3.94E-14	2.61E-12
hsa-miR-204-5p	−3.385048334	4.99E-14	2.61E-12
hsa-miR-136-3p	−2.378717319	7.48E-14	3.52E-12
hsa-miR-378c	−2.529296096	9.57E-14	4.10E-12
hsa-miR-100-5p	−1.957154262	2.56E-13	9.26E-12
hsa-miR-30e-5p	−1.438413193	8.61E-13	2.90E-11
hsa-miR-29c-3p	−2.528703007	2.32E-12	7.28E-11
hsa-miR-99a-3p	−2.428873139	2.79E-12	8.20E-11
hsa-let-7c-5p	−2.56136633	3.70E-12	1.02E-10
hsa-miR-378a-5p	−1.993443856	9.76E-12	2.19E-10
hsa-miR-381-3p	−2.957588852	2.68E-11	5.75E-10
hsa-miR-101-5p	−1.711233075	4.18E-11	8.56E-10
hsa-miR-139-3p	−2.07771688	5.79E-11	1.09E-09
hsa-miR-299-5p	−2.309271223	9.31E-11	1.68E-09
hsa-miR-125b-5p	−1.382091675	1.30E-10	2.18E-09
hsa-miR-125b-2-3p	−2.491649904	1.50E-10	2.36E-09

### GO and pathway analysis of DEGs

**Fig. 3:**
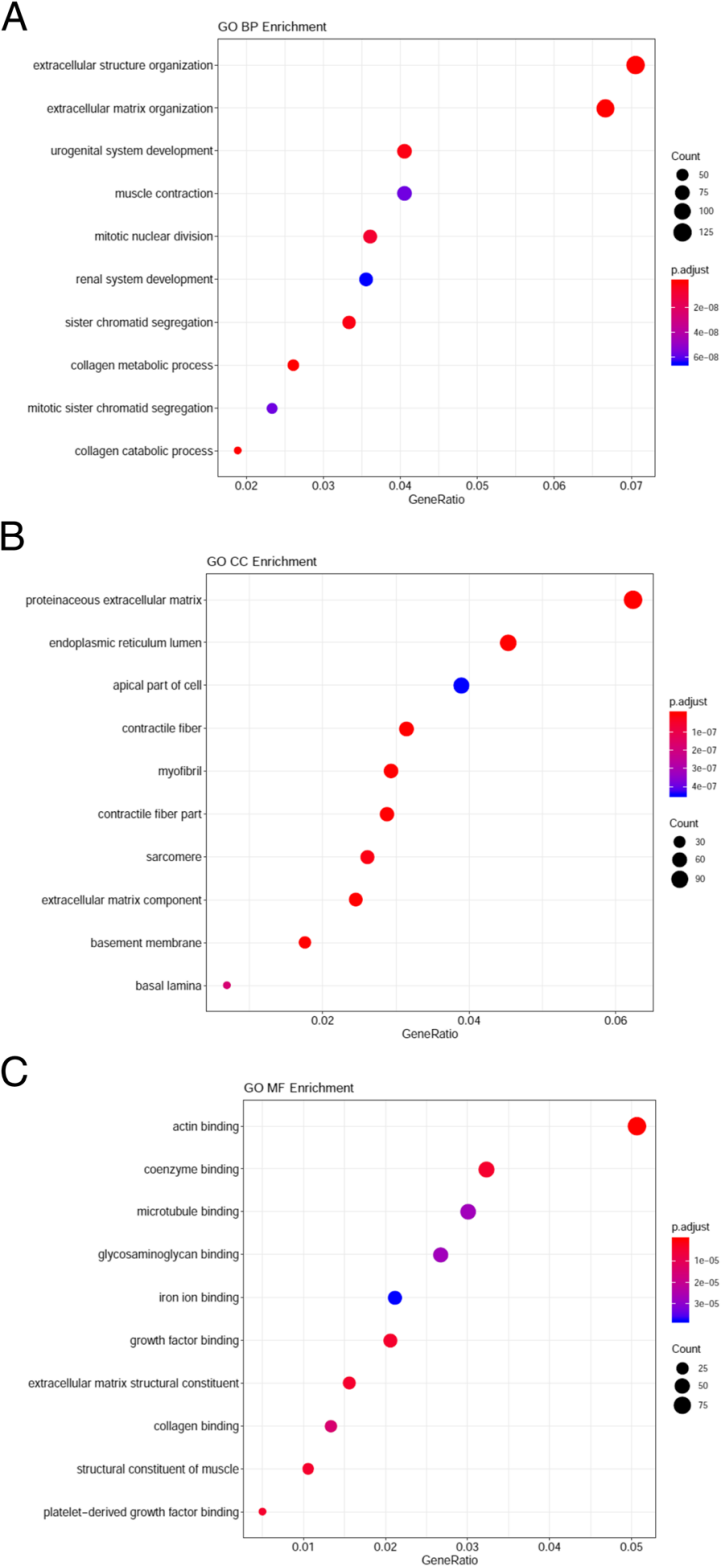
GO enrichment analysis of DEGs in SCCT. (Top 10). **a** Bubble Plot of BP. **b** Bubble Plot of CC. **c** Bubble Plot of MF

**Fig. 4:**
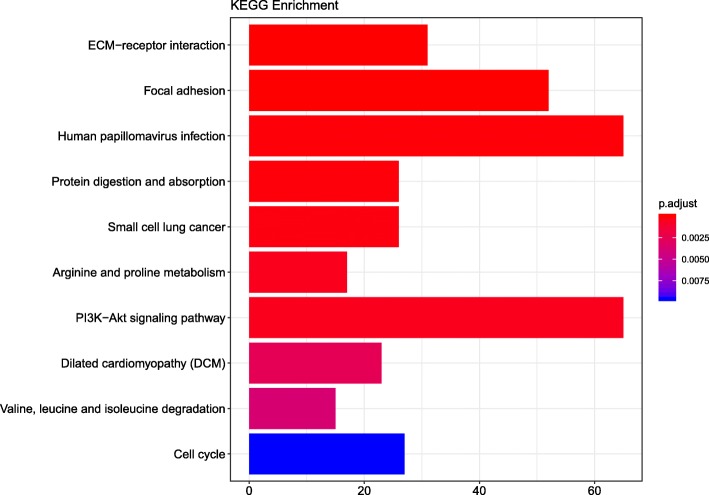
Top 10 enrichment of KEGG pathway analysis of DEGs

GO analysis results showed that changes in biological processes (BP) of DEGs were significantly enriched in extracellular structure organization, extracellular matrix organization, urogenital system development, muscle contraction, collagen metabolic process, mitotic nuclear division, renal system development, collagen catabolic process, sister chromatid segregation, and collagen metabolic process (Fig. 3a). Changes in cell component (CC) of DEGs were mainly enriched in proteinaceous extracellular matrix, endoplasmic reticulum lumen, apical part of cell, contractile fiber, myofibril, contractile fiber part, sarcomere, extracellular matrix component, basement membrane, basal lamina (Fig. 3b). Changes in molecular function (MF) were mainly enriched in actin binding, growth factor binding, coenzyme binding, microtubule binding, iron ion binding, glycosaminoglycan binding, collagen binding, structural constituent of muscle, extracellular matrix structural constituent, platelet−derived growth factor binding (Fig. 3c). KEGG pathway analysis revealed that the DEGs were mainly enriched in focal adhesion, human papillomavirus infection, ECM − receptor interaction, protein digestion and absorption, small cell lung cancer, arginine and proline metabolism, PI3K-Akt signaling pathway, dilated cardiomyopathy (DCM), valine, leucine and isoleucine degradation, cell cycle (Fig. 4).

### Survival analysis with the DEGs and DEmRNAs

**Fig. 5:**
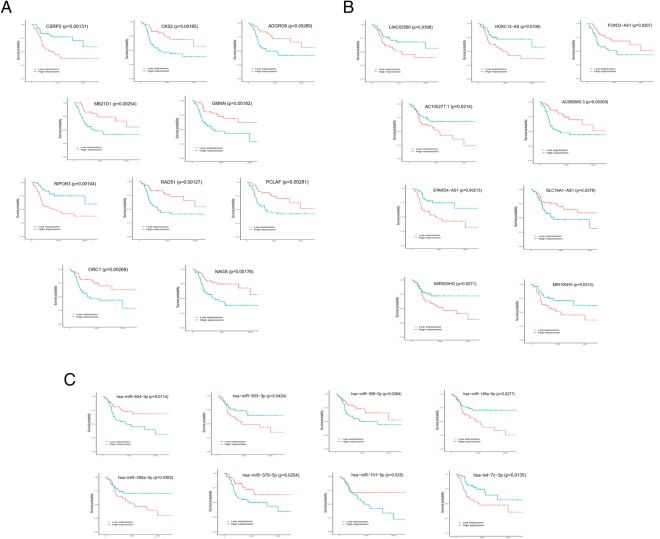
Kaplan-Meier survival curves for mRNAs (**a**), lncRNAs (**b**), and miRNAs (**c**) associated with overall survival (Top 10)

We studied the association of the DEGs and DEmRNAs with patient’ survival to identify the key genes and mRNAs that were related to the prognosis of patients with SCCT. We identified 10 mRNAs (CSRP2, CKS2, ADGRG6, MB21D1, GMNN, RIPOR3, RAD51, PCLAF, ORC1, NAGS), 9 lncRNAs (LINC02560, HOXC13 − AS, FOXD2 − AS1, AC105277.1, AC099850.3, STARD4 − AS1, SLC16A1 − AS1, MIR503HG, MIR100HG) and 8 miRNAs (miR − 654, miR − 503, miR − 450a, miR − 379, miR − 369, miR − 190a, miR − 101, let−7c) that were significantly differentially expressed in the survival analyses (Fig. 5a-c).

### Construction and analysis of the lncRNA-miRNA-mRNA ceRNA network

**Fig. 6:**
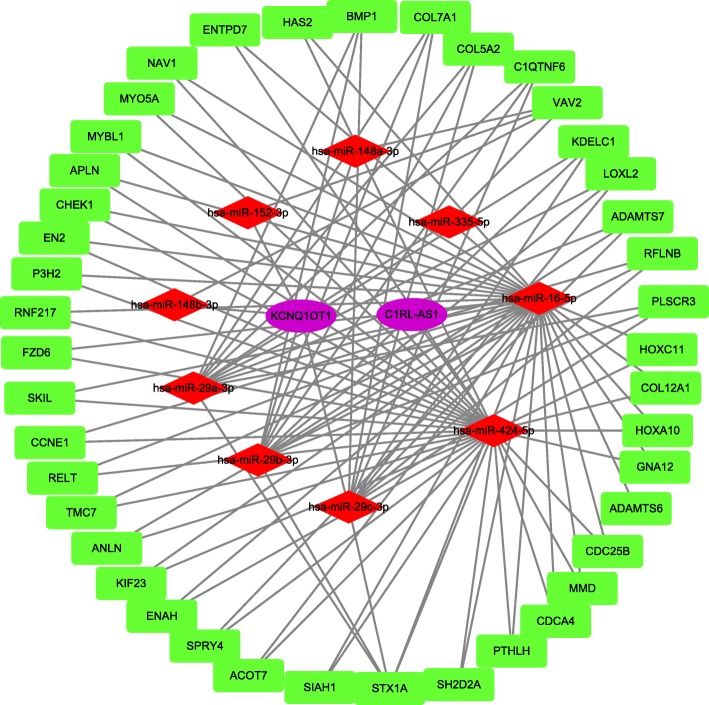
The lncRNA-miRNA-mRNA Competing endogenous RNA network. The rectangles indicate mRNAs in light green, ellipses represent lncRNAs in light purple and diamonds represent miRNAs in light red

**Fig. 7:**
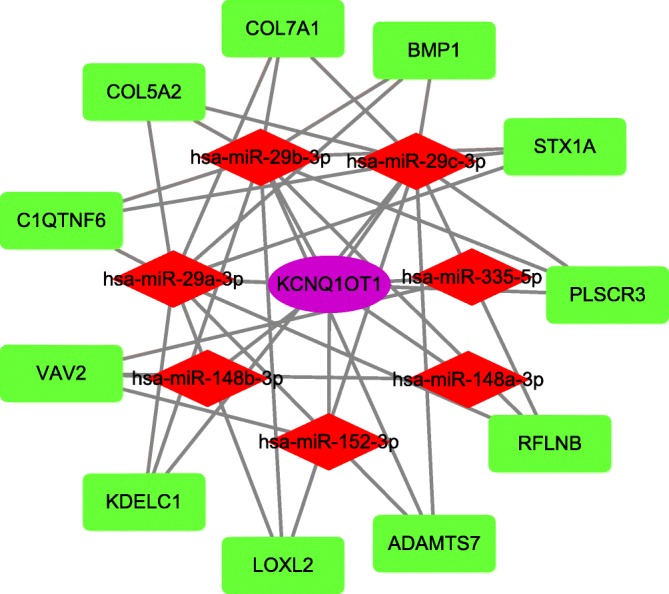
The sub-network of lncRNA KCNQ1OT1 and the GO terms interaction network. The lncRNA KCNQ1OT1 sub-network. The rectangles indicate mRNAs in light green, ellipses represent lncRNAs in light purple and diamonds represent miRNAs in light red

We built the ceRNA network on the basis of the miRNA, lncRNA, and mRNA the expression profiles in patients with SCCT. In total, 27 miRNA nodes, 53 mRNA nodes, 6 lncRNA nodes, and 152 edges were identified as differentially expressed profiles. The network is showed in Fig. 6. It is well known that lncRNAs and mRNAs have co-expression patterns in ceRNA networks. Thus, we chose a hub lncRNA (degree> 5, Additional file 4: Table S2) and its linked mRNAs and miRNAs in the triple global network and then reconstructed the sub-network. As shown in Fig. 7, the lncRNA KCNQ1OT1-miRNA-mRNA sub-network was composed of 1 lncRNA node, 7 miRNA nodes, 11 mRNA nodes, and 41 edges.

## Discussion

SCCT, a major type of HNSCC, is a refractory cancer under current therapeutics [33]. Studies have demonstrated that lncRNAs regulate gene expression through a variety of pathways, contributing to tumorigenesis and tumor metastasis [34, 35]. The ceRNA hypothesis proposes a new regulatory mechanism mediated by lncRNAs that are used as endogenous miRNA sponges [19, 36–38]. In this study, we found the genes and mRNAs that were differentially expressed between normal and tumor tissue. Through GO and KEGG analyses, we further analyzed the pathways and functions in which the DEGs are involved. The GO biological processes results suggested that specific genes may be concentrated in several process areas, such as extracellular structures, muscle contraction, and mitotic nuclear division. Some of the annotated pathways have been shown to be associated with cancer in previous studies. PI3K-Akt signaling is involved in cell proliferation and growth as well as down-regulating cell apoptosis [39]. Recent preclinical and clinical studies of highly selective agents that target various regulators of the mammalian cell cycle have demonstrated cell-cycle arrest in models of human cancer [40]. Through survival analysis, we identified 10 mRNAs (CSRP2, CKS2, ADGRG6, MB21D1, GMNN, RIPOR3, RAD51, PCLAF, ORC1, NAGS), 9 lncRNAs (LINC02560, HOXC13 − AS, FOXD2 − AS1, AC105277.1, AC099850.3, STARD4 − AS1, SLC16A1 − AS1, MIR503HG, MIR100HG) and 8 miRNAs (miR − 654, miR − 503, miR − 450a, miR − 379, miR − 369, miR − 190a, miR − 101, let−7c) that were significantly related to the overall survival of patients with SCCT. Next, by using bioinformatics tools, we builted a ceRNA network with SCCT-specific miRNA and lncRNA expression and selected the hub lncRNA KCNQ1OT1 to construct a sub-network.

KCNQ1OT1, also known as KCNQ1 overlapping transcript 1, is an imprinted antisense lncRNA in the KCNQ1 locus [41, 42]. Early studies have shown that KCNQ1OT1 is up-regulated and involved in the tumorigenesis of breast cancer and hepatocellular carcinoma [43, 44]. Zhang et al. found that KCNQ1OT1 could induce SCCT cell growth and inhibit the sensitivity of the tumor to cisplatin [27]. Previous studies have shown that KCNQ1OT1 acts as an oncogene and plays a key role in promoting SCCT cell growth and chemotherapy resistance.

## Conclusion

We constructed a SCCT-specific ceRNA network and chose a hub lncRNA for SCCT by bioinformatics analysis. To the best of our knowledge, only a limited number of studies have analyzed lncRNA obtained from large-scale samples. We provide a method for identifying potential lncRNA biomarkers. Furthermore, we found the ceRNA network in SCCT, which should help further our understanding of the mechanism underlying the pathogenesis of this disease.

## Additional files

Additional file 1: **Figure S1.** Boxplot of normalized RNA expression data (PDF 96 kb)

Additional file 2: **Figure S2.** Boxplot of normalized miRNA expression data (PDF 20 kb)

Additional file 3: **Table S1.** 118 SCCT patients clinical data (DOCX 25 kb)

Additional file 4: **Table S2.** The degree of ceRNA network. (DOCX 18 kb)

## Data Availability

All relevant data are within the manuscript.

## Abbreviations

BP: Biological processes
CC: Cell component
ceRNA: Competing endogenous RNA
DCM: Dilated cardiomyopathy
DEGs: Differentially expressed genes
EMT: Epithelial–mesenchymal transition
GO: Gene Ontology
HNSCC: Head and neck squamous cell carcinoma
HPV: Human papillomavirus
KEGG: Kyoto Encyclopedia of Genes and Genomes
lncRNA: long non-coding RNA
MF: Molecular function
miRNAs: microRNAs MREs MicroRNA response elements
ncRNA: non-coding RNA
SCCT: Squamous cell carcinoma of tongue
