# Supplementary figures and images for: Integrated analysis of lncRNA-miRNA-mRNA ceRNA network in squamous cell carcinoma of tongue
Source: BMC Cancer. 2019 Aug 7;19:779. doi: 10.1186/s12885-019-5983-8 (PMC6686570; doi:10.1186/s12885-019-5983-8)

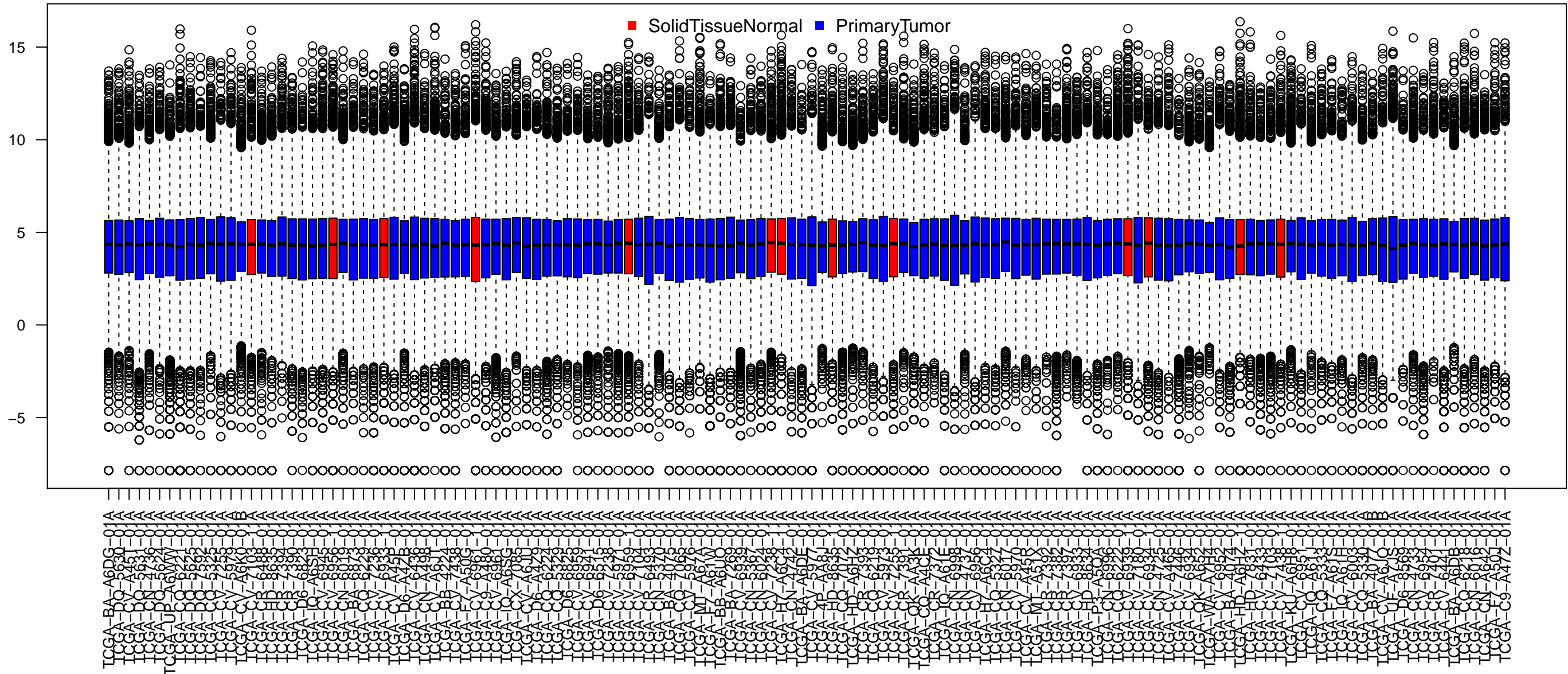

Supplement: Supplementary file 1 — Figure S1. Boxplot of normalized RNA expression data (PDF 96 kb) [file 12885_2019_5983_MOESM1_ESM.pdf]

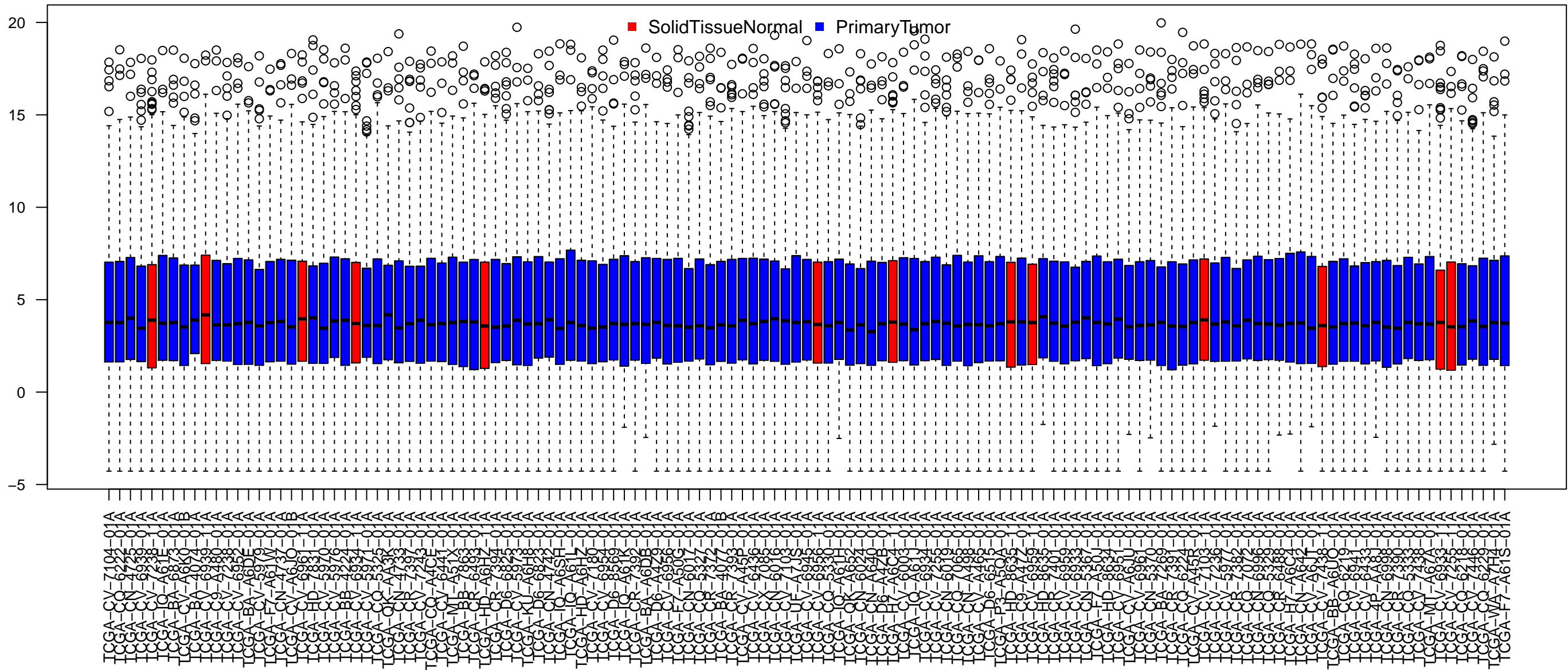

Supplement: Supplementary file 2 — Figure S2. Boxplot of normalized miRNA expression data (PDF 20 kb) [file 12885_2019_5983_MOESM2_ESM.pdf]
