# Supplementary material for: Integrated analysis of lncRNA-miRNA-mRNA ceRNA network in squamous cell carcinoma of tongue
Source: BMC Cancer. 2019 Aug 7;19:779. doi: 10.1186/s12885-019-5983-8 (PMC6686570; doi:10.1186/s12885-019-5983-8)
Supplement: Supplementary file 3 — Table S1. 118 SCCT patients clinical data (DOCX 25 kb) [file 12885_2019_5983_MOESM3_ESM.docx]

**Table S1.** 118 SCCT patients clinical data

| ethnicity | primary_diagnosis | tumor_  stage | vital_status | Tumr grade | tissue_or_organ_of_  origin |
| --- | --- | --- | --- | --- | --- |
| not reported | Squamous cell carcinoma, NOS | stage iva | dead | not reported | Tongue, NOS |
| not reported | Squamous cell carcinoma, NOS | stage iva | dead | not reported | Tongue, NOS |
| not reported | Squamous cell carcinoma, NOS | stage iva | dead | not reported | Tongue, NOS |
| not hispanic or latino | Squamous cell carcinoma, NOS | stage i | dead | not reported | Tongue, NOS |
| not hispanic or latino | Squamous cell carcinoma, NOS | stage i | dead | not reported | Tongue, NOS |
| not hispanic or latino | Squamous cell carcinoma, NOS | stage i | dead | not reported | Tongue, NOS |
| not hispanic or latino | Squamous cell carcinoma, NOS | stage ii | dead | not reported | Tongue, NOS |
| not hispanic or latino | Squamous cell carcinoma, NOS | stage ii | dead | not reported | Tongue, NOS |
| not hispanic or latino | Squamous cell carcinoma, NOS | stage ii | dead | not reported | Tongue, NOS |
| not hispanic or latino | Squamous cell carcinoma, NOS | stage ii | dead | not reported | Tongue, NOS |
| not hispanic or latino | Squamous cell carcinoma, NOS | stage iii | dead | not reported | Tongue, NOS |
| not hispanic or latino | Squamous cell carcinoma, NOS | stage iii | dead | not reported | Tongue, NOS |
| not hispanic or latino | Squamous cell carcinoma, NOS | stage iii | dead | not reported | Tongue, NOS |
| not hispanic or latino | Squamous cell carcinoma, NOS | stage iii | dead | not reported | Tongue, NOS |
| not hispanic or latino | Squamous cell carcinoma, keratinizing, NOS | stage iii | dead | not reported | Tongue, NOS |
| not hispanic or latino | Squamous cell carcinoma, NOS | stage iii | dead | not reported | Tongue, NOS |
| not hispanic or latino | Squamous cell carcinoma, NOS | stage iva | dead | not reported | Tongue, NOS |
| not hispanic or latino | Squamous cell carcinoma, NOS | stage iva | dead | not reported | Tongue, NOS |
| not hispanic or latino | Squamous cell carcinoma, NOS | stage iva | dead | not reported | Tongue, NOS |
| not hispanic or latino | Squamous cell carcinoma, NOS | stage iva | dead | not reported | Tongue, NOS |
| not hispanic or latino | Squamous cell carcinoma, NOS | stage iva | dead | not reported | Tongue, NOS |
| not hispanic or latino | Squamous cell carcinoma, keratinizing, NOS | stage iva | dead | not reported | Tongue, NOS |
| not hispanic or latino | Squamous cell carcinoma, NOS | stage iva | dead | not reported | Tongue, NOS |
| not hispanic or latino | Squamous cell carcinoma, NOS | stage iva | dead | not reported | Tongue, NOS |
| not hispanic or latino | Squamous cell carcinoma, NOS | stage iva | dead | not reported | Tongue, NOS |
| not hispanic or latino | Squamous cell carcinoma, keratinizing, NOS | stage iva | dead | not reported | Tongue, NOS |
| not hispanic or latino | Squamous cell carcinoma, NOS | stage iva | dead | not reported | Tongue, NOS |
| not hispanic or latino | Squamous cell carcinoma, NOS | stage iva | dead | not reported | Tongue, NOS |
| not hispanic or latino | Squamous cell carcinoma, NOS | stage iva | dead | not reported | Tongue, NOS |
| not hispanic or latino | Squamous cell carcinoma, NOS | stage iva | dead | not reported | Tongue, NOS |
| not hispanic or latino | Squamous cell carcinoma, NOS | stage iva | dead | not reported | Tongue, NOS |
| not hispanic or latino | Squamous cell carcinoma, NOS | stage iva | dead | not reported | Tongue, NOS |
| not hispanic or latino | Squamous cell carcinoma, NOS | stage iva | dead | not reported | Tongue, NOS |
| not hispanic or latino | Squamous cell carcinoma, NOS | stage iva | dead | not reported | Tongue, NOS |
| not hispanic or latino | Squamous cell carcinoma, NOS | stage iva | dead | not reported | Tongue, NOS |
| not hispanic or latino | Squamous cell carcinoma, NOS | stage iva | dead | not reported | Tongue, NOS |
| not hispanic or latino | Squamous cell carcinoma, NOS | stage iva | dead | not reported | Tongue, NOS |
| not hispanic or latino | Squamous cell carcinoma, NOS | stage iva | dead | not reported | Tongue, NOS |
| not hispanic or latino | Squamous cell carcinoma, NOS | stage iva | dead | not reported | Tongue, NOS |
| not hispanic or latino | Squamous cell carcinoma, NOS | stage iva | dead | not reported | Tongue, NOS |
| not hispanic or latino | Squamous cell carcinoma, keratinizing, NOS | stage iva | dead | not reported | Tongue, NOS |
| not hispanic or latino | Squamous cell carcinoma, NOS | stage ivb | dead | not reported | Tongue, NOS |
| hispanic or latino | Squamous cell carcinoma, NOS | stage iii | dead | not reported | Tongue, NOS |
| hispanic or latino | Squamous cell carcinoma, NOS | stage iii | dead | not reported | Tongue, NOS |
| hispanic or latino | Squamous cell carcinoma, NOS | stage iii | dead | not reported | Tongue, NOS |
| hispanic or latino | Squamous cell carcinoma, NOS | stage iva | dead | not reported | Tongue, NOS |
| not reported | Squamous cell carcinoma, NOS | stage i | alive | not reported | Tongue, NOS |
| not reported | Squamous cell carcinoma, keratinizing, NOS | stage ii | alive | not reported | Tongue, NOS |
| not hispanic or latino | Squamous cell carcinoma, NOS | stage i | alive | not reported | Tongue, NOS |
| not hispanic or latino | Squamous cell carcinoma, NOS | stage i | alive | not reported | Tongue, NOS |
| not hispanic or latino | Squamous cell carcinoma, NOS | stage i | alive | not reported | Tongue, NOS |
| not hispanic or latino | Squamous cell carcinoma, NOS | stage i | alive | not reported | Tongue, NOS |
| not hispanic or latino | Squamous cell carcinoma, NOS | stage i | alive | not reported | Tongue, NOS |
| not hispanic or latino | Squamous cell carcinoma, NOS | stage i | alive | not reported | Tongue, NOS |
| not hispanic or latino | Squamous cell carcinoma, NOS | stage i | alive | not reported | Tongue, NOS |
| not hispanic or latino | Squamous cell carcinoma, NOS | stage i | alive | not reported | Tongue, NOS |
| not hispanic or latino | Squamous cell carcinoma, keratinizing, NOS | stage i | alive | not reported | Tongue, NOS |
| not hispanic or latino | Squamous cell carcinoma, NOS | stage ii | alive | not reported | Tongue, NOS |
| not hispanic or latino | Squamous cell carcinoma, NOS | stage ii | alive | not reported | Tongue, NOS |
| not hispanic or latino | Squamous cell carcinoma, keratinizing, NOS | stage ii | alive | not reported | Tongue, NOS |
| not hispanic or latino | Squamous cell carcinoma, keratinizing, NOS | stage ii | alive | not reported | Tongue, NOS |
| not hispanic or latino | Squamous cell carcinoma, NOS | stage ii | alive | not reported | Tongue, NOS |
| not hispanic or latino | Squamous cell carcinoma, NOS | stage ii | alive | not reported | Tongue, NOS |
| not hispanic or latino | Squamous cell carcinoma, NOS | stage ii | alive | not reported | Tongue, NOS |
| not hispanic or latino | Squamous cell carcinoma, NOS | stage ii | alive | not reported | Tongue, NOS |
| not hispanic or latino | Squamous cell carcinoma, NOS | stage ii | alive | not reported | Tongue, NOS |
| not hispanic or latino | Squamous cell carcinoma, keratinizing, NOS | stage ii | alive | not reported | Tongue, NOS |
| not hispanic or latino | Squamous cell carcinoma, NOS | stage ii | alive | not reported | Tongue, NOS |
| not hispanic or latino | Squamous cell carcinoma, NOS | stage ii | alive | not reported | Tongue, NOS |
| not hispanic or latino | Squamous cell carcinoma, NOS | stage ii | alive | not reported | Tongue, NOS |
| not hispanic or latino | Squamous cell carcinoma, NOS | stage iii | alive | not reported | Tongue, NOS |
| not hispanic or latino | Squamous cell carcinoma, NOS | stage iii | alive | not reported | Tongue, NOS |
| not hispanic or latino | Squamous cell carcinoma, keratinizing, NOS | stage iii | alive | not reported | Tongue, NOS |
| not hispanic or latino | Squamous cell carcinoma, NOS | stage iii | alive | not reported | Tongue, NOS |
| not hispanic or latino | Squamous cell carcinoma, NOS | stage iii | alive | not reported | Tongue, NOS |
| not hispanic or latino | Squamous cell carcinoma, NOS | stage iii | alive | not reported | Tongue, NOS |
| not hispanic or latino | Squamous cell carcinoma, keratinizing, NOS | stage iii | alive | not reported | Tongue, NOS |
| not hispanic or latino | Squamous cell carcinoma, NOS | stage iii | alive | not reported | Tongue, NOS |
| not hispanic or latino | Squamous cell carcinoma, keratinizing, NOS | stage iii | alive | not reported | Tongue, NOS |
| not hispanic or latino | Squamous cell carcinoma, NOS | stage iii | alive | not reported | Tongue, NOS |
| not hispanic or latino | Squamous cell carcinoma, NOS | stage iii | alive | not reported | Tongue, NOS |
| not hispanic or latino | Squamous cell carcinoma, NOS | stage iii | alive | not reported | Tongue, NOS |
| not hispanic or latino | Squamous cell carcinoma, NOS | stage iii | alive | not reported | Tongue, NOS |
| not hispanic or latino | Squamous cell carcinoma, NOS | stage iii | alive | not reported | Tongue, NOS |
| not hispanic or latino | Squamous cell carcinoma, NOS | stage iii | alive | not reported | Tongue, NOS |
| not hispanic or latino | Squamous cell carcinoma, NOS | stage iii | alive | not reported | Tongue, NOS |
| not hispanic or latino | Squamous cell carcinoma, NOS | stage iva | alive | not reported | Tongue, NOS |
| not hispanic or latino | Squamous cell carcinoma, NOS | stage iva | alive | not reported | Tongue, NOS |
| not hispanic or latino | Squamous cell carcinoma, NOS | stage iva | alive | not reported | Tongue, NOS |
| not hispanic or latino | Squamous cell carcinoma, NOS | stage iva | alive | not reported | Tongue, NOS |
| not hispanic or latino | Squamous cell carcinoma, NOS | stage iva | alive | not reported | Tongue, NOS |
| not hispanic or latino | Squamous cell carcinoma, NOS | stage iva | alive | not reported | Tongue, NOS |
| not hispanic or latino | Squamous cell carcinoma, NOS | stage iva | alive | not reported | Tongue, NOS |
| not hispanic or latino | Squamous cell carcinoma, NOS | stage iva | alive | not reported | Tongue, NOS |
| not hispanic or latino | Squamous cell carcinoma, NOS | stage iva | alive | not reported | Tongue, NOS |
| not hispanic or latino | Squamous cell carcinoma, keratinizing, NOS | stage iva | alive | not reported | Tongue, NOS |
| not hispanic or latino | Squamous cell carcinoma, NOS | stage iva | alive | not reported | Tongue, NOS |
| not hispanic or latino | Squamous cell carcinoma, NOS | stage iva | alive | not reported | Tongue, NOS |
| not hispanic or latino | Squamous cell carcinoma, NOS | stage iva | alive | not reported | Tongue, NOS |
| not hispanic or latino | Squamous cell carcinoma, NOS | stage iva | alive | not reported | Tongue, NOS |
| not hispanic or latino | Squamous cell carcinoma, keratinizing, NOS | stage iva | alive | not reported | Tongue, NOS |
| not hispanic or latino | Squamous cell carcinoma, NOS | stage iva | alive | not reported | Tongue, NOS |
| not hispanic or latino | Squamous cell carcinoma, NOS | stage iva | alive | not reported | Tongue, NOS |
| not hispanic or latino | Squamous cell carcinoma, NOS | stage iva | alive | not reported | Tongue, NOS |
| not hispanic or latino | Squamous cell carcinoma, NOS | stage iva | alive | not reported | Tongue, NOS |
| not hispanic or latino | Squamous cell carcinoma, keratinizing, NOS | stage iva | alive | not reported | Tongue, NOS |
| not hispanic or latino | Squamous cell carcinoma, NOS | stage iva | alive | not reported | Tongue, NOS |
| not hispanic or latino | Squamous cell carcinoma, keratinizing, NOS | stage iva | alive | not reported | Tongue, NOS |
| not hispanic or latino | Squamous cell carcinoma, NOS | stage iva | alive | not reported | Tongue, NOS |
| not hispanic or latino | Squamous cell carcinoma, NOS | stage iva | alive | not reported | Tongue, NOS |
| not hispanic or latino | Squamous cell carcinoma, keratinizing, NOS | stage iva | alive | not reported | Tongue, NOS |
| not hispanic or latino | Squamous cell carcinoma, NOS | stage iva | alive | not reported | Tongue, NOS |
| not hispanic or latino | Squamous cell carcinoma, NOS | stage ivb | alive | not reported | Tongue, NOS |
| hispanic or latino | Squamous cell carcinoma, keratinizing, NOS | stage ii | alive | not reported | Tongue, NOS |
| hispanic or latino | Squamous cell carcinoma, keratinizing, NOS | stage ii | alive | not reported | Tongue, NOS |
| hispanic or latino | Squamous cell carcinoma, keratinizing, NOS | stage iii | alive | not reported | Tongue, NOS |
| hispanic or latino | Squamous cell carcinoma, NOS | stage iii | alive | not reported | Tongue, NOS |
| hispanic or latino | Squamous cell carcinoma, NOS | stage iva | alive | not reported | Tongue, NOS |
