# Supplementary material for: Integrated analysis of lncRNA-miRNA-mRNA ceRNA network in squamous cell carcinoma of tongue
Source: BMC Cancer. 2019 Aug 7;19:779. doi: 10.1186/s12885-019-5983-8 (PMC6686570; doi:10.1186/s12885-019-5983-8)
Supplement: Supplementary file 4 — Table S2. The degree of ceRNA network. (DOCX 18 kb) [file 12885_2019_5983_MOESM4_ESM.docx]

**Table S2.** The degree of ceRNA network.

| degree.layout | gene | name | type |
| --- | --- | --- | --- |
| 33 | hsa-miR-16-5p | hsa-miR-16-5p | mir |
| 32 | hsa-miR-424-5p | hsa-miR-424-5p | mir |
| 11 | hsa-miR-29a-3p | hsa-miR-29a-3p | mir |
| 11 | hsa-miR-29b-3p | hsa-miR-29b-3p | mir |
| 11 | hsa-miR-29c-3p | hsa-miR-29c-3p | mir |
| 7 | ENSG00000269821 | KCNQ1OT1 | lnc |
| 5 | ENSG00000106089 | STX1A | pc |
| 4 | ENSG00000160293 | VAV2 | pc |
| 3 | ENSG00000187838 | PLSCR3 | pc |
| 3 | ENSG00000183688 | RFLNB | pc |
| 3 | ENSG00000136378 | ADAMTS7 | pc |
| 3 | ENSG00000134013 | LOXL2 | pc |
| 3 | ENSG00000134901 | KDELC1 | pc |
| 3 | ENSG00000133466 | C1QTNF6 | pc |
| 3 | ENSG00000204262 | COL5A2 | pc |
| 3 | ENSG00000114270 | COL7A1 | pc |
| 3 | ENSG00000168487 | BMP1 | pc |
| 2 | ENSG00000170961 | HAS2 | pc |
| 2 | ENSG00000198018 | ENTPD7 | pc |
| 2 | ENSG00000134369 | NAV1 | pc |
| 2 | ENSG00000197535 | MYO5A | pc |
| 2 | ENSG00000185697 | MYBL1 | pc |
| 2 | ENSG00000171388 | APLN | pc |
| 2 | ENSG00000149554 | CHEK1 | pc |
| 2 | ENSG00000164778 | EN2 | pc |
| 2 | ENSG00000090530 | P3H2 | pc |
| 2 | ENSG00000146373 | RNF217 | pc |
| 2 | ENSG00000164930 | FZD6 | pc |
| 2 | ENSG00000136603 | SKIL | pc |
| 2 | ENSG00000105173 | CCNE1 | pc |
| 2 | ENSG00000054967 | RELT | pc |
| 2 | ENSG00000170537 | TMC7 | pc |
| 2 | ENSG00000011426 | ANLN | pc |
| 2 | ENSG00000137807 | KIF23 | pc |
| 2 | ENSG00000154380 | ENAH | pc |
| 2 | ENSG00000187678 | SPRY4 | pc |
| 2 | ENSG00000097021 | ACOT7 | pc |
| 2 | ENSG00000196470 | SIAH1 | pc |
| 2 | ENSG00000027869 | SH2D2A | pc |
| 2 | ENSG00000087494 | PTHLH | pc |
| 2 | ENSG00000170779 | CDCA4 | pc |
| 2 | ENSG00000108960 | MMD | pc |
| 2 | ENSG00000101224 | CDC25B | pc |
| 2 | ENSG00000146535 | GNA12 | pc |
| 2 | ENSG00000253293 | HOXA10 | pc |
| 2 | ENSG00000111799 | COL12A1 | pc |
| 2 | ENSG00000123388 | HOXC11 | pc |
| 2 | hsa-miR-335-5p | hsa-miR-335-5p | mir |
| 2 | hsa-miR-148a-3p | hsa-miR-148a-3p | mir |
| 2 | hsa-miR-152-3p | hsa-miR-152-3p | mir |
| 2 | hsa-miR-148b-3p | hsa-miR-148b-3p | mir |
| 2 | ENSG00000205885 | C1RL-AS1 | lnc |
| 1 | ENSG00000049192 | ADAMTS6 | pc |
